# Feasibility study for the use of self‐collected nasal swabs to identify pathogens among participants of a population‐based surveillance system for acute respiratory infections (GrippeWeb‐Plus)—Germany, 2016

**DOI:** 10.1111/irv.12644

**Published:** 2019-03-29

**Authors:** Joana M. Haussig, Angelina Targosz, Susanne Engelhart, Michael Herzhoff, Kerstin Prahm, Silke Buda, Andreas Nitsche, Walter Haas, Udo Buchholz

**Affiliations:** ^1^ Department of Infectious Disease Epidemiology Robert Koch Institute Berlin Germany; ^2^ Postgraduate Training for Applied Epidemiology (PAE) Robert Koch Institute Berlin Germany; ^3^ European Centre for Disease Prevention and Control (ECDC) Stockholm Sweden; ^4^ Centre for Biological Threats and Special Pathogens Robert Koch Institute Berlin Germany

**Keywords:** epidemiological monitoring, human, influenza, patient generated health data, public health surveillance, respiratory tract infections, self-swabbing

## Abstract

**Background:**

Internet‐based participatory surveillance systems, such as the German GrippeWeb, monitor the frequency of acute respiratory illnesses on population level. In order to interpret syndromic information better, we devised a microbiological feasibility study (GrippeWeb‐Plus) to test whether self‐collection of anterior nasal swabs is operationally possible, acceptable for participants and can yield valid data.

**Methods:**

We recruited 103 GrippeWeb participants (73 adults and 30 children) and provided them with a kit, instructions and a questionnaire for each sample. In the first half of 2016, participants took an anterior nasal swab and sent it to the Robert Koch Institute whenever an acute respiratory illness occurred. Reporting of illnesses through the GrippeWeb platform continued as usual. We analysed swabs for the presence of human c‐myc‐DNA and 22 viral and bacterial pathogens. After the study, we sent participants an evaluation questionnaire. We analysed timeliness, completeness, acceptability and validity.

**Results:**

One hundred and two participants submitted 225 analysable swabs. Ninety per cent of swabs were taken within 3 days of symptom onset. Eighty‐nine per cent of swabs had a corresponding reported illness in the GrippeWeb system. Ninety‐nine per cent of adults and 96% of children would be willing to participate in a self‐swabbing scheme for a longer period. All swabs contained c‐myc‐DNA. In 119 swabs, we identified any of 14 viruses but no bacteria. The positivity rate of influenza was similar to that in the German physician sentinel.

**Conclusion:**

Self‐collection of anterior nasal swabs proofed to be feasible, was well accepted by participants, gave valid results and was an informative adjunct to syndromic data.

## 1 INTRODUCTION

To estimate the burden of disease on population level due to acute respiratory infections (ARI), influenza‐like illness (ILI) or influenza in particular Internet‐based, participatory surveillance systems have been set up in the last 15 years in several countries within and outside of Europe.^1^, ^2^ In Germany, a system called GrippeWeb has been launched in 2011.^6^ Advantages of GrippeWeb include that it works year‐round and generates representative data in all age groups. Moreover, as GrippeWeb also collects information if a person with ARI consults a physician for his or her illness GrippeWeb data on “ARI with ensuing consultation of a primary health care provider” could be successfully compared and cross‐validated with data on “physician consultations due to ARI” generated by the German sentinel physician network “Arbeitsgemeinschaft Influenza” (AGI).^6^

Most physician sentinel networks use a second pillar, namely microbiological analyses of respiratory samples taken from primary care patients, to assist in the interpretation of syndromic data derived from the same patient population. In contrast, population‐based, participatory surveillance systems typically lack this kind of information. Thus, there is little up‐to‐date information what kind of pathogens cause respiratory infections that may or may not lead to physician consultations. However, longitudinal information on the type of pathogens would be helpful to interpret syndromic data, focus on specific risk groups, calculate (pathogen‐specific) burden of disease estimates, support therapeutic decisions (eg, regarding the use or non‐use of antibiotic therapy) and, finally, guide decisions on the development of vaccines that could reduce disease of responsible pathogens. To keep complexity and cost low, the very nature of these participatory surveillance instruments calls for a simple mechanism of respiratory sample collection by participants themselves. Self‐collection may be more acceptable when nasal or particularly anterior nasal swabs can be used. While in clinical practice pharyngeal or nasopharyngeal swabs are taken frequently for upper respiratory illness, it has been shown that for influenza and other respiratory viruses, nasal swabs are at least equally sensitive if not superior to pharyngeal specimens.^7^, ^8^ Furthermore, Akmatov demonstrated that self‐collected and medical staff‐collected nasal swabs were equivalent in acceptance and the capability to detect pathogens.^11^ We therefore designed a feasibility study (“GrippeWeb‐Plus”) to test if self‐collection of anterior nasal swabs is logistically possible, acceptable for participating adults and children, and if it yields valid microbiological results that are capable to complement syndromic surveillance data.

## 2 METHODS

### 2.1 The GrippeWeb system

We have previously described the GrippeWeb system in detail.^1^ Briefly, GrippeWeb runs throughout the year and every person residing in Germany who is at least 14 years old can register. Parents can report for their children aged 13 years or younger. Upon registration, participants answer questions on demographic variables, lifetime physician‐diagnosed chronic conditions, smoking, household size, daily occupation and main mode of transportation used. Participants receive weekly emails summarizing the GrippeWeb results published on the system's website and reminding them to complete the weekly online questionnaire. In this questionnaire, participants are asked whether they have experienced the onset of a new respiratory illness during the previous week. If the participant has had a respiratory illness with new symptom onset, participants were additionally asked to report the date of onset, selected symptoms from a short list (cough, sore throat, fever and coryza), if they have consulted a physician because of the illness and whether they have been able to continue their usual daily occupation. Influenza vaccination is recorded weekly during the winter season. Participants can report weekly answers retrospectively up to a maximum of four weeks. Reported illnesses are recorded in the participant's personal password‐protected online diary and can be accessed online at any time. In February 2016, about 4500‐5000 participants from all over Germany delivered their weekly report, ensuring a broad geographical representation.

### 2.2 Study population and recruitment

For the purpose of this study, we had bought multiplex PCR tests RespiFinder® 2SMART (Pathofinder, Maastricht, NL) (see section “Laboratory analysis”). Since the number of tests was limited, we aimed for a maximum of 300 samples to be processed. For the inclusion of participants, we took into account the following parameters: (a) each participant should send in a baseline swab to proof that he/she is capable to take a swab and to lower any inhibitions; (b) an adult:child ratio of 2:1; (c) the number of ARI per adult and children of 1‐1.5 and 1.5‐2.5 per year^6^; (d) an estimated 50% response rate for invited participants; and (e) to leave some space so that test kits would not run out while the study is still ongoing. In addition, we wished to also include a smaller number of RKI employees as a “highly motivated” comparison group.

We conducted the study in the first half of 2016 and aimed to recruit adult participants (≥18 years) registered and actively participating in GrippeWeb. We defined “active participation” as reporting at least two thirds of the possible weekly notifications in the 18 weeks before week 40/2015. Among these, we selected 137 GrippeWeb participants randomly and invited them by email to participate in GrippeWeb‐Plus. Invited participants could also enrol their children in GrippeWeb‐Plus if they were registered in GrippeWeb. Upon expression of interest, we mailed participants by post‐additional information for the GrippeWeb‐Plus feasibility study as well as consent forms. Informed consent forms had to be signed by every participant to be enrolled in the study. Consent forms for children had to be signed by both parents with right of custody. In addition, consent forms for children aged 14‐17 years had to be signed by the children themselves. We aimed to enrol 80 adults and 40 children. In addition to the randomly selected participants, we asked 11 employees of the Robert Koch Institute (RKI), who already participated in GrippeWeb, to also participate in this study. This group was thought to serve as a (highly motivated) comparison group.

### 2.3 Operational definitions

GrippeWeb defines an acute respiratory illness (ARI) as a subjectively reported respiratory illness with new onset of reported fever or cough or sore throat. Influenza‐like illness is defined as a subjectively reported respiratory illness with new onset of reported fever together with cough or sore throat. Therefore, all ILI are a subset of all ARI. Notified illnesses that state coryza as the only symptom are not categorized as ARI. In this study, a “symptomatic” patient is defined as a patient who submitted a swab when he or she subjectively felt to have new onset of a respiratory illness.

### 2.4 Study design

After signing the informed consent forms, each participating household received a kit including an information leaflet explaining the procedures, an instruction on how to take an anterior nasal swab, three swabs per adult and four swabs per child with corresponding numbers of vials with virus transport medium, personalized stickers with swab numbers and a short paper‐based questionnaire. The questionnaire was used to record symptoms when a sample is taken, date of onset, date of swabbing, if a physician was consulted because of the illness, whether the participant has been able to continue his/her usual daily occupation despite the illness, how the self‐collection of the sample was perceived and if an injury had occurred during swabbing. Participants were asked to continue with the online (anonymous) reporting of illnesses to GrippeWeb. Using the global unique identifier (GUID) and a self‐given nickname of each participant, we were able to collate data from GrippeWeb and GrippeWeb‐Plus.

Collection of swabs started in January 2016. Participants were asked to provide a swab at the beginning of the study, regardless if they had symptoms or not, in order to test the study logistics and analyse the presence of pathogens in asymptomatic participants. During the study period, between January and July 2016, we asked participants to collect anterior nasal swabs whenever they or their participating children had symptoms of a respiratory infection. Participants were reminded through the weekly email to take swabs when they experience a respiratory illness. We asked participants to take the swabs not later than three days after symptom onset, but we accepted swabs in the analysis when they were taken less than 10 days after symptom onset. We requested participants to fill in the paper‐based symptom questionnaire and label these with provided stickers containing the GUID, nickname and a unique sample number. An identical sticker was used to label the corresponding sample tube. We also provided pre‐paid packaging material so that swabs and questionnaires could be sent by mail to the collaborating laboratory at the RKI.

Individual laboratory results of their swabs were fed back to each participant through their personal GrippeWeb diary webpage that can be individually accessed at any time using the login of the participant.

After the study period had ended, we sent a paper‐based evaluation questionnaire to the participants to assess if they thought the study procedures were clear and acceptable, and their perception of self‐swabbing. For analysis purposes, answers stating “don't know” and missing answers were excluded from the denominator.

### 2.5 Laboratory analysis

Following the purification protocol of viral nucleic acids for fluid samples, we extracted nucleic acids from 200 µl of the swab medium (Flocked Swab with UTM, Fa. COPAN Flock Technologies srl., Brescia, Italy) with the QIAamp Min Elute Virus Spin Kit (Cat. No. 57704, Qiagen, Hilden, Germany). The internal control of the analysis kit was added directly into the AL buffer.

The remaining native samples of the patients and the extracted RNA/DNA were stored at −80°C before and after the analysis. A pooled medium sample from three humans who had tested negative previously was used as a negative extraction control.

Self‐collected swabs were analysed for 22 viral and bacterial pathogens using the RespiFinder® 2SMART (Pathofinder, Maastricht, NL) according to the manufacturer's protocol. Tested pathogens included influenza A, influenza A(H1N1)pdm09, influenza B, parainfluenza‐1, parainfluenza‐2, parainfluenza‐3, parainfluenza‐4, RSV‐A, RSV‐B, human metapneumovirus, rhinovirus/enterovirus, bocavirus (type 1), adenovirus, coronavirus NL63, coronavirus HKU1, coronavirus 229E, coronavirus OC43, *Mycoplasma pneumoniae*, *Chlamydophila pneumoniae*, *Legionella pneumophila* and *Bordetella pertussis*. A(H3N2) was not detected specifically; we assumed that influenza A‐positive samples were influenza A(H3N2) if they were negative for influenza A(H1N1)pdm09.

Because the laboratory analysis included notifiable diseases (according to the “Protection against Infection Act” (www.gesetze-im-internet.de/ifsg/), namely influenza*, *whooping cough (*Bordetella pertussis*)*, *legionellosis (*Legionella pneumophila*) and in the Free State of Saxony also respiratory syncytial virus (RSV), we notified the responsible local public health department whenever one of these pathogens was identified. Participants were informed about our obligation to report in the consent form.

To ensure that swabs included human cells, we tested each swab for the presence of human c‐myc‐DNA. Samples that yielded an equivocal result were repeated.

### 2.6 Data analysis

We entered data in Microsoft Excel 2010 (Redmond, WA, USA) and analysed them with Stata version 14 (Stata Corporation, College Station, TX, United States). Descriptive analysis included data on recruitment, the number of submitted swabs and time intervals between symptom onset, day of swabbing and arrival of the swab in the laboratory.

To evaluate completeness of swabbing, we compared results from the group of randomly selected participants with that among participating staff members of the RKI. To do this, we first merged the GrippeWeb and GrippeWeb‐Plus databases and then compared (a) the proportion of swabs where a respiratory illness was reported and (b) the proportion of respiratory illnesses that were reported online where a swab was taken. Proportions were analysed using the chi‐square test of homogeneity. We assessed acceptability in two ways: we analysed self‐reported side effects at the time when each swab was taken (as documented in the symptom questionnaire) as well as self‐reported judgement of acceptability in the final evaluation questionnaire.

To test internal validity of the samples, we examined (a) the proportion of variables filled out in the data of the forms submitted by the participants; (b) the number of participants who dropped out; (c) completeness of swabbing (see above); (d) the proportion of evaluation forms returned; and (e) if samples contained the human c‐myc‐gene which can only be found in human cells. To test external validity**,** we compared virological results with that of the virological surveillance system of the physician sentinel AGI.^12^ Because AGI physicians take swabs from patients with ILI, we compared positivity rates (PRs) of influenza and rhinoviruses also among GrippeWeb‐Plus participants presenting with ILI. Of note, the ILI definition in the AGI includes fever + one systemic symptom such as headache or myalgia + one respiratory symptom and differs therefore from the ILI definition in GrippeWeb. Since the proportion of samples among children was very similar compared to that in the virological surveillance system in the AGI during the same weeks (42% among GrippeWeb‐Plus samples; 40% among AGI samples) and for specific pathogens numbers became very small, no age‐adjusted PR was calculated. For the period of influenza circulation (PIC), we used the definition provided by the AGI (week 2‐week 15 [2016]).

To analyse pathogen results, we analysed the frequency of identified pathogens, co‐infections and stratified results for symptomatic and asymptomatic participants, as well as for children and adults.

### 2.7 Data protection and ethics approval

GrippeWeb‐Plus was carried out according to the German legislation on data protection. The GrippeWeb‐Plus procedures were approved by the German Federal Commissioner for Data Protection and Freedom of Information (ID: III‐401/008#0072). The GrippeWeb‐Plus study was approved by the Ethics Committee of the Charité, Ethikausschuss 2 am Campus Virchow‐Klinikum (ID: EA2/066/15). All participants gave written informed consent before taking part.

## 3 RESULTS

### 3.1 Recruitment

**Figure 1:**
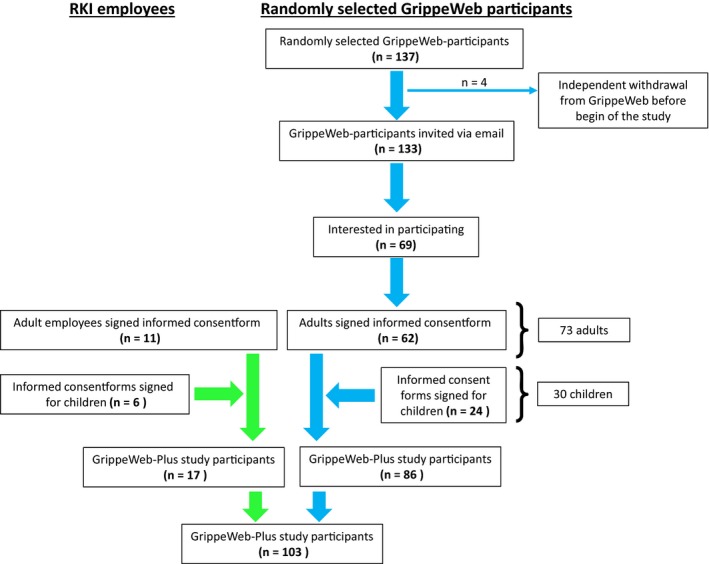
Recruitment of study participants. Right side (blue arrows): randomly selected GrippeWeb participants and their children, left side (red arrows): RKI employees and their children. GrippeWeb‐Plus study, January‐July 2016, Germany

We selected 137 GrippeWeb participants for the study. Among those, four had terminated their participation in GrippeWeb before we had tried to contact them and were excluded (Figure 1). Thus, we invited 133 via email to participate. Of those, 69 (52%) expressed interest in participating, and finally, 62 (47%) signed the consent form. These participants enrolled 24 (63%) of their children in the study. Furthermore, the 11 RKI employees signed up six (100%) of their children. In total, this led to 73 adults and 30 children participating in the GrippeWeb‐Plus study (Figure 1).

### 3.2 Submitted swabs

Participants took swabs between 17 January 2016 and 13 July 2016 (week 2‐week 28). A total of 227 swabs were submitted by 102 (99%) of 103 participants. Two swabs were excluded as the date of symptom onset preceded the date of swabbing by several weeks. Among the remaining 225 swabs, 151 swabs were from symptomatic participants, 58 swabs were from asymptomatic participants, and 16 swabs were submitted without information on symptoms. Swabs from participants with symptoms arrived at the laboratory between week 5 and week 30/2016. The mean number of submitted swabs per symptomatic patient with at least one submitted swab was 1.4 per adult and 2.4 per child.

### 3.3 Timeliness

After symptom onset, 90% of swabs were taken within 3 days and 88% arrived at the laboratory within 10 days. The mean and median time between symptom onset and swabbing was 1.9 days and 2 days, respectively.

### 3.4 Completeness

The proportion of swabs of participants with symptoms (n = 151) where also a respiratory illness was reported through GrippeWeb was 89% (134/151) for the exact same week and increased to 94% (142/151) if one week earlier or later was allowed. Randomly selected GrippeWeb participants reported an illness through GrippeWeb in 96% (119/124), more often than participating RKI employees (85% [23/27]; *P*‐value = 0.03). The proportion of GrippeWeb reports notifying a respiratory illness during the study period where also a swab was submitted was 61% (119/196) among randomly selected GrippeWeb participants, again more often than among participating RKI employees (43% (23/54); *P*‐value = 0.02). Frequency of taking a swab was independent of illness severity.

### 3.5 Acceptability

Information about the experience during swabbing was contained in 208 (92%) of 225 reports accompanying the swabs. In 33 (16%), participants reported that it was unpleasant and in 3 (1%; two 6‐year‐old children and one adult), participants answered “yes” to the question if an injury occurred. In the comment box of the report of the swab taken from the first child, the parent indicated that the child had expressed some aching, for the second child no comment was provided. However, three further swabs were submitted from that child in the course of the study. The third report (from the adult) provided information that taking the swab had led to some temporary irritation which was commented as “not bad.” In the final evaluation questionnaire, the parents of the two children answered that no injury had occurred and the adult participant did not provide a final evaluation questionnaire.

The final evaluation questionnaire was answered by 101 (98%) participants (Table 1). All adult participants and 86% of children indicated that self‐swabbing was either unproblematic or briefly unpleasant (*P*‐value < 0.01), none reported an injury. Ninety‐nine per cent of adults and 96% of children would be willing to participate in a self‐swabbing scheme for a longer period of time. All adult participants found the general study procedure acceptable, all but one adult stated that the study procedure was explained comprehensibly, and all adults found the information on how to self‐swab comprehensible.

**Table 1:** Results of post‐study evaluation questionnaire among study participants. GrippeWeb‐Plus study, Germany, January‐July 2016

Question asked	Age group	Answer	Randomly selected participant (%)	RKI participants (%)	Total (%)	*P*‐value for comparison of randomly selected vs RKI participants	*P*‐value for comparison of children vs adults
Answered evaluation questionnaires	Adults		61/63 (97)	11/11 (100)	72/74 (97)	0.55	0.37
	Children		23/23 (100)	6/6 (100)	29/29 (100)	Undefined	
	Total		84/86 (98)	17/17 (100)	101/103 (98)		0.53
Perception of self‐swabbing?	Adults	Unproblematic	50/61 (82)	11/11 (100)	61/72 (85)	Undefined	<0.01
		Only briefly unpleasant	11/61 (18)	0/11 (0.0)	11/72 (15)		
		Longer unpleasant	0/61 (0.0)	0/11 (0.0)	0/72 (0.0)		
		Refusal	0/61 (0.0)	0/11 (0.0)	0/72 (0.0)		
	Children	Unproblematic	9/23 (39)	2/6 (33)	11/29 (38)	0.04	
		Only briefly unpleasant	12/23 (52)	2/6 (33)	14/29 (48)		
		Longer unpleasant	0/23 (0.0)	2/6 (33)	2/29 (6.9)		
		Refusal	2/23 (8.7)	0/6 (0.0)	2/29 (6.9)		
Did an injury occur?	Adults	Yes	0/61 (0.0)	0/11 (0.0)	0/72 (0.0)	Undefined	Undefined
		No	61/61 (100)	11/11 (100)	72/72 (100)		
	Children	Yes	0/23 (0.0)	0/6 (0.0)	0/29 (0.0)	Undefined	
		No	23/23 (100)	6/6 (100)	29/29 (100)		
Willing to participate in self‐swabbing for longer time?	Adults	Yes	58/59 (98)	11/11 (100)	69/70 (99)	0.66	0.48
		No	1/59 (1.7)	0/11 (0.0)	1/70 (1.4)		
	Children	Yes	20/21 (95)	6/6 (100)	26/27 (96)	0.59	
		No	1/21 (4.8)	0/6 (0.0)	1/27 (3.7)		
Did you find it interesting to know which pathogens were detected?	Adults	Yes	46/55 (84)	10/11 (91)	56/66 (85)	0.54	0.03
		No	9/55 (16)	1/11 (9.1)	10/66 (14)		
	Children	Yes	22/22 (100)	6/6 (100)	28/28 (100)	Undefined	
		No	0/22 (0.0)	0/6 (0.0)	0/28 (0.0)		
General study procedure acceptable?	Adults	Yes	61/61 (100)	11/11 (100)	72/72 (100)	Undefined	NA
		No	0/61 (0.0)	0/11 (0.0)	0/72 (0.0)		
	Children	*not asked*				NA	
Information on study procedure comprehensible and sufficient?	Adults	yes	60/61 (98)	11/11 (100)	71/72 (99)	0.67	NA
		no	1/61 (1.6)	0/11 (0.0)	1/72 (1.4)		
	Children	*not asked*				NA	
Information on self‐swabbing comprehensible ?	Adults	yes	61/61 (100)	11/11 (100)	72/72 (100)	Undefined	NA
		no	0/61 (0.0)	0/11 (0.0)	0/72 (0.0)		
	Children	*not asked*				NA	
Were testing results easy to find?^a^	Adults	Yes	45/51 (88)	9/11 (82)	54/62 (87)	0.56	NA
		No	6/51 (12)	2/11 (18)	8/62 (13)		
	Children	*not asked*				NA	

NA = not applicable.

a Referring to the personal password protected diary at the GrippeWeb Internet page.

### 3.6 Internal validity

The proportion of variables filled out was 92% in the forms that were submitted for swabs from asymptomatic participants and 99% from symptomatic participants. Two children dropped out of the study. For completeness of swabbing, see above under “completeness.” The evaluation form was returned by 99% of adult participants, and only one form was missing.

### 3.7 Laboratory results

We identified the c‐myc‐gene in all analysed samples.

In 96 of 119 positive swabs (81%) (regardless of symptom information), we detected one pathogen, in 22 swabs (18%) two different pathogens and in one swab (0.8%) three different pathogens, accounting for a total of 143 pathogen detections. Overall, we identified 14 different viruses, but no bacteria (Table 2). Among the 143 virus detections, rhinovirus/enterovirus (42%) and coronavirus NL63/HKU1 (17%) were identified most frequently, followed by bocavirus (10%) and influenza viruses (7.0% influenza A(H1N1)pdm09 and 4.9% influenza B) (Tab. 1). Grouping by phylogenetic family picornaviruses (rhino‐/enteroviruses) dominated with 42%, followed by coronaviruses with 23%, orthomyxoviruses (influenza A and influenza B) with 12% and bocaviruses with 10%.

**Table 2:** Detected pathogens among all swabs of participants with single, double and triple infections, stratified by symptomatic/asymptomatic participants, Germany, January‐July 2016

Pathogen	All swabs		Symptomatic		Asymptomatic		Unknown	
	n	%	n	%	n	%	n	%
Rhino‐/enterovirus	60	42	54	42	4	40	2	40
CoV NL63/HKU1	25	17	20	16	3	30	2	40
Bocavirus	15	10	12	9.4	2	20	1	20
INV A(H1N1)pdm09	10	7.0	10	7.8	0	0	0	0
INV B	7	4.9	7	5.5	0	0	0	0
CoV 229E	5	3.5	5	3.9	0	0	0	0
RSV A	5	3.5	5	3.9	0	0	0	0
hMPV	5	3.5	5	3.9	0	0	0	0
CoV OC43	3	2.1	3	2.3	0	0	0	0
Adenovirus	2	1.4	1	0.8	1	10	0	0
RSV B	2	1.4	2	1.6	0	0	0	0
PIV1	2	1.4	2	1.6	0	0	0	0
PIV 2	1	0.7	1	0.8	0	0	0	0
PIV 3	1	0.7	1	0.8	0	0	0	0
PIV4	0	0	0	0	0	0	0	0
INV A(H3N2)	0	0	0	0	0	0	0	0
*Mycoplasma pneumoniae*	0	0	0	0	0	0	0	0
*Legionella pneumophila*	0	0	0	0	0	0	0	0
*Bordetella pertussis*	0	0	0	0	0	0	0	0
*Chlamydophila pneumoniae*	0	0	0	0	0	0	0	0
Total	143	100	128	100	10	100	5	100

CoV = coronavirus; hMPV = human metapneumovirus; INV = influenza; PIV = parainfluenza virus; RSV = respiratory syncytial virus.

We detected at least one pathogen in 107 of 151 swabs (71%) among symptomatic participants, in 8 of 58 swabs (14%) among asymptomatic participants, and in 4 of 16 swabs (25%) where symptom information was missing (Table 3). Among the three participant groups, the proportion of positive swabs where only one pathogen was detected varied little (range: 75%‐81%; Table 3).

**Table 3:** Positivity rates of swabs by presence of symptoms among participants, Germany, January‐July 2016

Swabs (n = 225) among…	Swab positivity		Number of pathogens detected		
	Negative	Positive (%)	1 (%)	2 (%)	3 (%)
Participants with symptoms (n = 151)	44	107 (71)	87 (81)	19 (18)	1 (1)
Participants without symptoms (n = 58)	50	8 (14)	6 (75)	2 (25)	0 (0)
Participants with no information about symptoms (n = 16)	12	4 (25)	3 (75)	1 (25)	0 (0)

**Figure 2:**
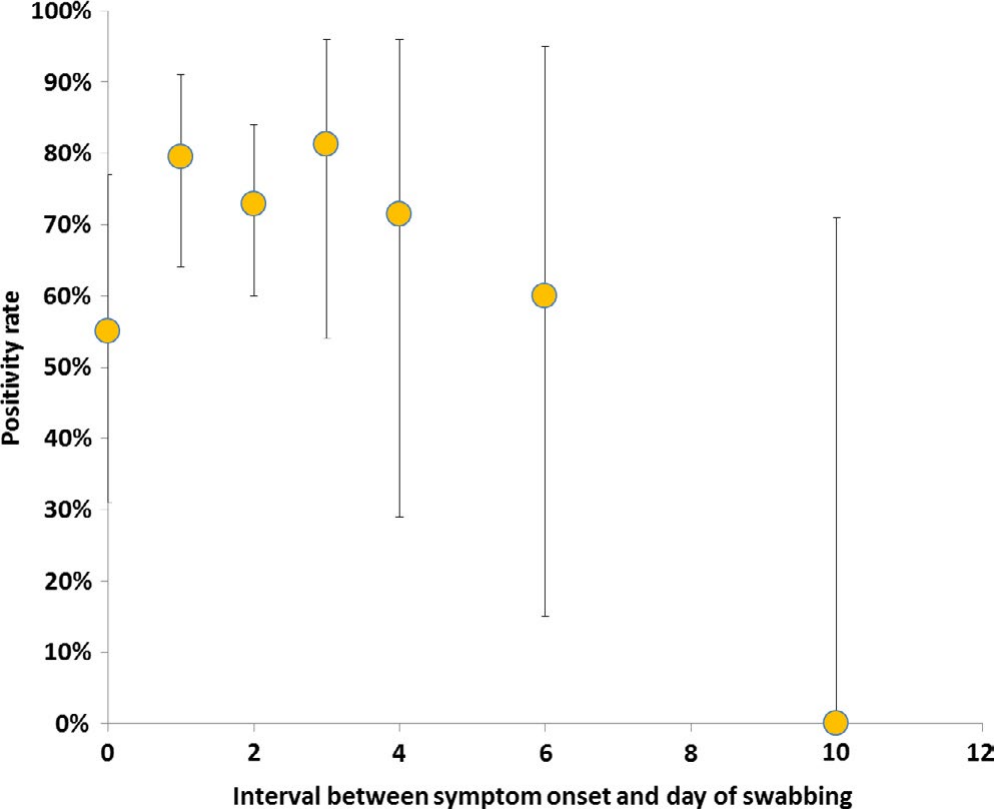
Positivity rate by interval between symptom onset and day of swabbing; data point for 6 d is pooled from days 5 to 7, January‐July 2016, Germany

Overall, the PR of swabs of participants with symptoms was 71%. PR was independent of the amount of c‐myc‐DNA identified. The PR by interval from symptom onset until day of swabbing varied between 55% and 81% when the swab was taken between 0 and 6 days (Figure 2). Between 1 day and 4 days, it was between 71% and 81%, respectively. Confidence intervals from day 4 onwards were large due to small numbers.

**Figure 3:**
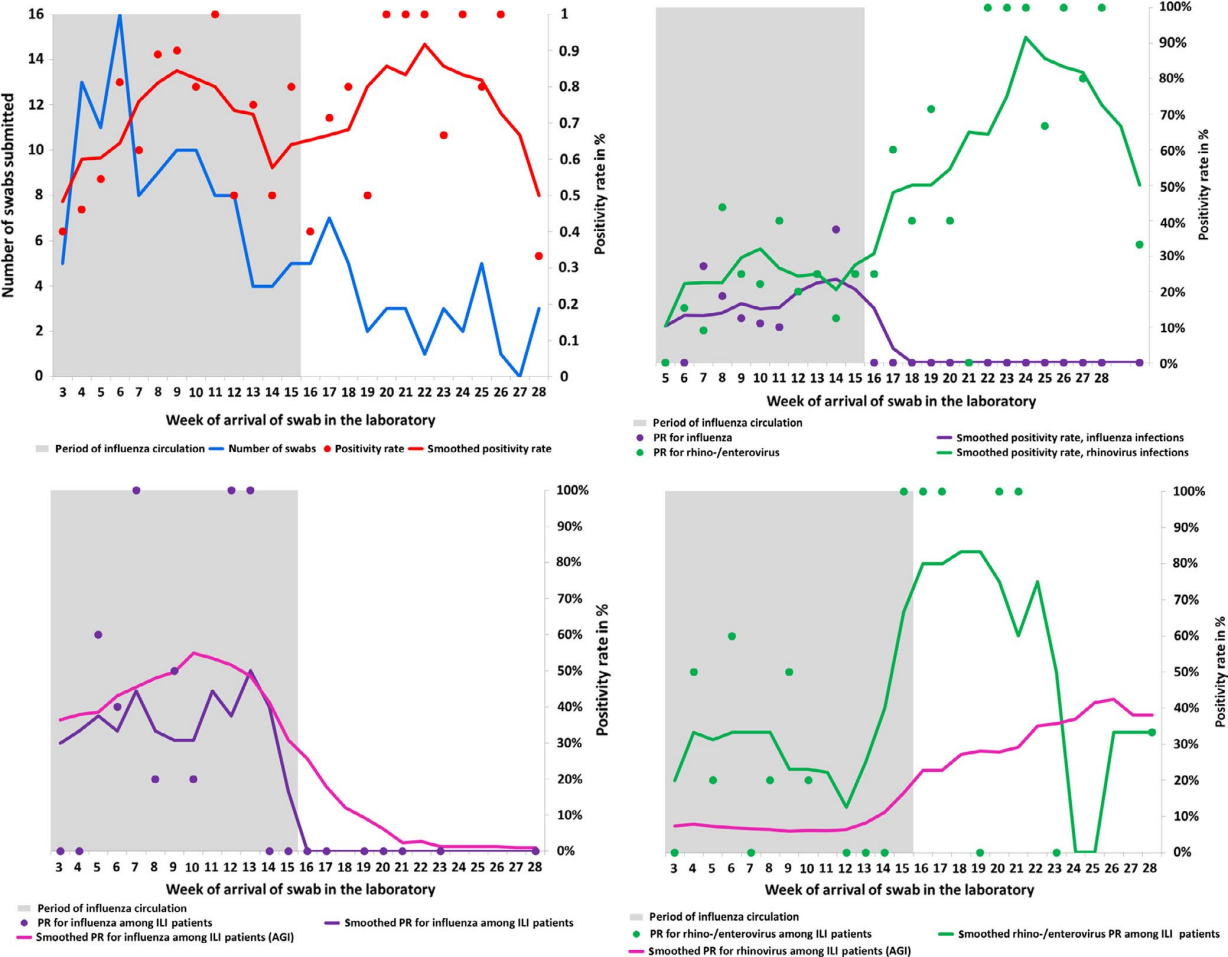
Upper left panel: number of swabs and positivity rate (PR) of any pathogen by week of arrival in the laboratory among symptomatic participants of the GrippeWeb‐Plus study; upper right: influenza and rhino‐/enterovirus PR; lower left: influenza PR among GrippeWeb‐Plus participants with influenza‐like illness (ILI) and influenza PR among patients of the German physician sentinel (AGI); lower right: rhino‐/enterovirus PR among GrippeWeb‐Plus participants with ILI and rhinovirus PR among patients of the AGI. PR was smoothed to guide the eye; GrippeWeb‐Plus study, January‐July 2016, Germany

Although PR varied by week of symptom onset between 0% and 100%, it reached 50% in most weeks and was not statistically significantly different during the PIC (Figure 3; upper left).

#### 3.7.1 PR for influenza and rhino‐/enterovirus among all symptomatic participants

**Figure 4:**
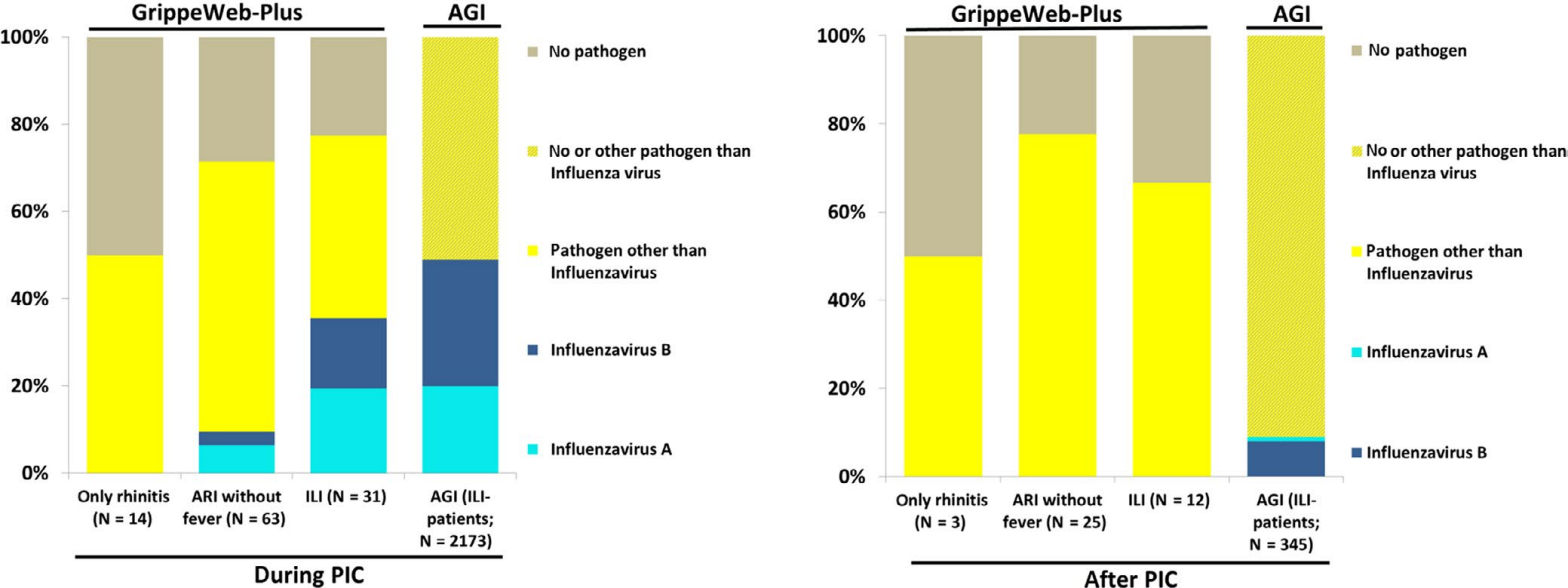
Detected pathogens by syndrome during the period of influenza circulation (left panel) and after the period of influenza circulation (right panel). ARI without fever (GrippeWeb‐Plus) = acute respiratory infection includes illnesses with cough or sore throat, but NOT fever; ILI = influenza‐like illness (GrippeWeb‐Plus) includes illnesses with fever and (cough or sore throat); ILI (AGI definition) = fever + 1 systemic symptom (eg, headache, muscle pain) + 1 respiratory symptom (eg, cough). GrippeWeb‐Plus study, January‐July 2016, Germany

During the PIC, PR for influenza virus among all symptomatic participants was between 10% and 20% (Figure 4, upper right panel) and dropped to 0 in the weeks after the PIC. In contrast, while during the PIC the PR for rhino‐/enteroviruses among all symptomatic participants was similar to influenza, it rose substantially in the 10 weeks thereafter (Figure 4, upper right panel).

#### 3.7.2 PR for influenza and rhino‐/enterovirus among participants with ILI and comparison with ILI patients in the AGI

Among participants with ILI, the weekly PR for influenza rose to approximately 35%‐50% during the PIC (compare Figure 3, upper right with lower left panel) and was similar to the PR among AGI patients with ILI (Figure 3, lower left panel). PR for rhino‐/enterovirus among participants with ILI was at best slightly higher than that among all symptomatic participants during the PIC (compare Figure 3, upper right with lower right panel) and rose after the end of the PIC; however, numbers were small. PR for rhino‐/enterovirus among participants with ILI could not be compared exactly with that among ILI patients in the AGI because in the AGI specimens are tested for rhinovirus only. Nevertheless, if both curves are held side‐by‐side, PR among GrippeWeb participants with ILI appears to be systematically higher by proximately 20 percentage points (Figure 3, lower right panel).

#### 3.7.3 PR for influenza vs non‐influenza viruses by syndrome and during/after PIC

During the PIC, influenza A and influenza B were detected in one swab (1.7%) and two swabs (3.4%; in total 5.1%), respectively, among participants with ARI without fever, and in nine swabs (24%) and five swabs (14%; in total 38%), respectively, among swabs from participants with ILI (Figure 4, left panel). In the same time period, the swabs taken from ILI patients of the AGI were positive in 20% for influenza A and in 29% for influenza B (in total 49%; Figure 4, left panel). After the PIC, no influenza virus was detected among swabs from GrippeWeb participants, and in 9% of swabs among AGI patients (Figure 4, right panel).

Coronavirus NL63/HKU1 was detected only during the PIC (18% [20/108]), but not after the PIC (0% [0/43]; *P*‐value = 0.002).

### 3.8 Children vs adults

**Figure 5:**
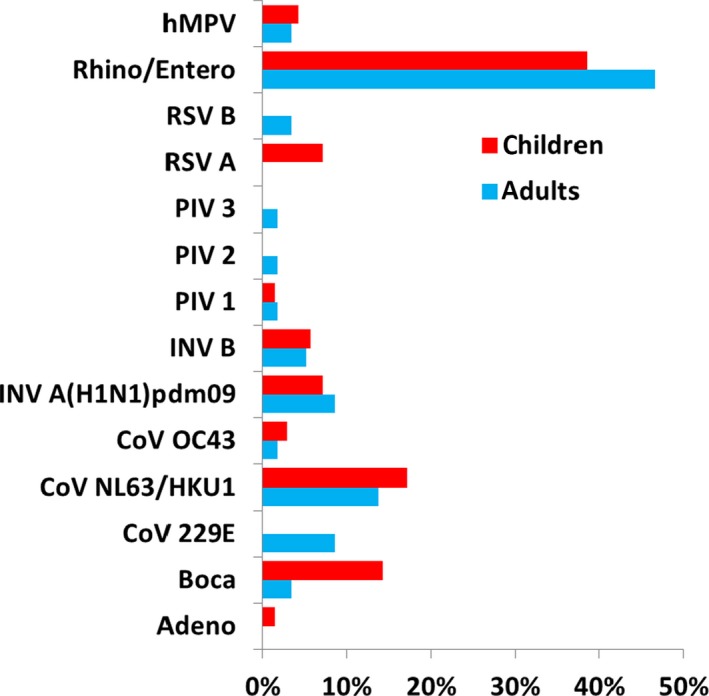
Pathogen distribution among symptomatic children (≤14 y) and adults (>14 y), January‐July 2016, Germany

Among swabs from asymptomatic participants, samples from children were as likely to yield a pathogen as those from adults (children: 20% [2/10]; adults: 13% [6/47]). In contrast, among symptomatic participants, samples from children were more likely to harbour an identifiable pathogen than among swabs from adults (children: ((85% (53/62); adults: 62% (54/87); *P* = 0.002)). The pathogen distribution among symptomatic children and adults was not significantly different (*P*‐value = 0.08; Figure 5).

### 3.9 Co‐infections

In 23 of 119 positive swabs (19%), more than one pathogen was detected and 47 of 143 pathogen detections (33%) occurred within a co‐infection of two (22 times) or three viruses (once). Double or triple infections among 0‐4‐year‐old children represented 44% (12/27), among 5‐14‐year‐old children 17% (5/30) and among adults 10% (6/62) of positive samples (*P*‐value 0.003). Stratified by pathogen, the proportion of detections as double or triple infection varied widely among pathogens and ranged from 0% (CoV 229E, RSV‐B, PIV2) to 100% (PIV3; n = 1; Table 4). Bocaviruses were detected in 73% within a double or triple infection. Of the pathogen, most often detected (rhino‐/enterovirus) in 18% the patient was co‐infected with another pathogen. The co‐infection matrix in Table 5 shows how often which combination of pathogens was identified. Combinations of the three viruses that were detected most frequently (rhino‐/enterovirus, CoV NL63/HKU1 and bocavirus; Table 2), including one triple infection of these three viruses, contributed to 9 of 23 co‐infections (39%). Stratified by syndrome, co‐infections were not more frequent in more severe infections (“coryza only” < ARI without fever < ILI), also when analysis was restricted to swabs without influenza.

**Table 4:** Frequency and proportion of pathogens detected in double and triple infections regardless of symptoms, sorted by number of detections, Germany, January‐July 2016

Pathogen	Number of detections, n	Number of detections in co‐infections	
		n	%
Rhino/Entero	60	11	18
CoV NL63/HKU1	25	10	40
Boca	15	11	73
INV A(H1N1)pdm09	10	4	40
INV B	7	1	14
RSV A	5	3	60
hMPV	5	3	60
CoV 229E	5	0	0
CoV OC43	3	1	33
Adeno	2	1	50
PIV1	2	1	50
RSV B	2	0	0
PIV 3	1	1	100
PIV 2	1	0	0
PIV4	0	NA	NA
INV A(H3N2)	0	NA	NA

**Table 5:** Frequency of combinations of pathogens detected in double infections and one triple infection regardless of symptoms, Germany, January‐July 2016

	Rhino‐/enterovirus	CoV NL63/HKU1	Bocavirus	INV A(H1N1)pdm09	INV B	RSV A	hMPV	CoV OC43	Adenovirus	PIV1	PIV 3
Rhino‐/enterovirus											
CoV NL63/HKU1	3 + 1										
Bocavirus	3 + 1	2 + 1									
Influenza A(H1N1)pdm09	2		2								
Influenza B	1										
RSV A		1	2								
hMPV		2	1								
CoV OC43											
Adenovirus								1			
PIV1		1									
PIV 3	1										

The single triple infection is noted by “+1” (rhino‐/enterovirus; bocavirus; coronavirus NL63/HKU1).

## 4 DISCUSSION

We consider this feasibility study as successful as the participation and quality of samples were more than satisfactory and led to valid results. In more detail, (a) the willingness to participate in the study was substantial; (b) swabs were taken timely and were almost always accompanied by an online report via the GrippeWeb system; (c) self‐swabbing was mostly perceived as unproblematic and was well accepted, among children and adults alike, no injury occurred; (d) almost all participants would be willing to participate in a self‐swabbing scheme for a prolonged period of time; (e) the quality of swabs taken was good as they always contained DNA from human cells; (f) the PR for any pathogen was above 50% throughout the entire study period; (g) for ILI patients, PR for influenza was similar to that in the AGI. The most frequently detected pathogen were (in descending order) rhinovirus/enterovirus, coronaviruses, influenzavirus and bocavirus.

About half of the initially selected GrippeWeb participants agreed to participate in the study. Because the group was selected from participants with known interest or a recent high reporting rate in GrippeWeb, it is likely that this group was particularly motivated. However, because it is unlikely that disease illness rates differ substantially between more or less motivated participants and because it would be important to ensure reliable participation in a future microbiological surveillance scheme, future operational procedures should build on the positive experience with the method used in this feasibility study.

Work‐up of quality indicators of the swabs submitted revealed that all swabs contained the c‐myc‐gene which indicates that they were taken with enough thoroughness to contain cells from the anterior nose. Moreover, up to 94% of the swabs had a corresponding report through the online GrippeWeb platform. The proportion of swabs taken after a report had been submitted was not as high (61%). In comparison with data from the literature, this proportion was lower than that reported by Wenham (77%),^13^ but higher than that reported from Goff (43%).^14^ While no data were collected on the reasons why a swab was taken or not taken, taking a swab was not associated with symptom severity, so the simplest explanation would be just oversight; nevertheless, more detailed information would be useful in the interpretation of results. Finally, swabs were taken timely, and the mean and median delay from symptom onset to swabbing was 1.9 days and 2 days, respectively, both lower than that reported by Goff (mean 3.29 days) 14 and Elliot (mean 4 days).^15^

Similar to the experience made by other researchers, self‐collection of nasal swabs was well accepted.^11^, ^13^ The large majority of both adults and children would have been willing to participate in a self‐swabbing scheme for a longer period of time. Feedback of the laboratory results was welcomed and appreciated by study participants (Table 1), and it is likely that it has helped that participants adhered conscientiously to the study. We therefore recommend this type of individual feedback to participants. Lastly, in terms of (external) validity PR for influenza among GW participants with ILI was similar to that in the virological sentinel of the AGI.^12^ Although numbers were small, PR for rhino‐/enterovirus among GrippeWeb‐Plus participants with ILI appeared to be systematically higher than PR for rhinovirus among AGI ILI patients. This result suggests that analysing swabs for enterovirus in addition to rhinovirus may increase the yield of swabs with an identified pathogen in the AGI.

During this study, a large variety of viruses was detected. More than 70% of swabs from symptomatic participants yielded at least one pathogen, more than in studies with a similar diagnostic spectrum (36%^11^; 48%^16^). The viruses most frequently identified were rhino‐/enterovirus, coronaviruses, influenza viruses as well as bocavirus. Except for bocavirus which was not tested in other studies, this finding is in broad agreement with results published by Goff (USA, 2013/2014)^14^ and Plymouth (Sweden, 2001/2012).^16^ However, pathogens that are rather detectable in the lower respiratory tract, perhaps particularly bacteria, may not be detectable through this method. In general, the possibility to detect a pathogen by PCR depends on the specimen taken (eg, nose swab, throat swab) and the time point of swab collection after symptom onset.^7^, ^9^ Thus, not detecting a pathogen does not necessarily mean absence of the pathogen in the patient. In addition, it is possible that participants were infected by pathogens that were not tested for.

Roughly, one in five positive swabs contained more than one pathogen. In the case of single symptomatic infections, it is often difficult to say which of two or more pathogens caused the disease, or if both or all contributed. Because of the lack of association of co‐infections with severity of disease, it is generally more likely that just one of the multiple pathogens is causing the illness. Among the four pathogens most frequently detected (rhino‐/enterovirus, coronavirus NL63/HKU1, influenza viruses and bocavirus), their pathogenic role seems to differ. Among infections involving bocavirus, 73% occurred in the context of a co‐infection, whereas the former three are part of a co‐infection in less than 50%. This supports the notion that bocaviruses are hardly capable to cause illness on their own, but may be identifiable “on the side” when a co‐pathogen does.

In summary, this feasibility study showed good acceptance and adherence to the study procedure by both child and adult participants. There was no significant difference to a highly motivated reference group from our own institute. Participants were very willing to participate in a longer lasting swabbing scheme which could form the base for continued surveillance with the goal to understand better the pathogens leading to ARI on population level. Asymptomatic swabs were useful in the beginning to “practice” the study procedure, but would also be helpful to serve as a reference for the frequency of detection of individual pathogens to determine their importance in causing disease.

## DISCLAIMER

The views and opinions expressed herein are the authors’ own and do not necessarily state or reflect those of ECDC. ECDC is not responsible for the data and information collation and analysis and cannot be held liable for conclusions or opinions drawn.
